# Oligonucleotide Microarray Identifies Genes Differentially Expressed during Tumorigenesis of DMBA-Induced Pancreatic Cancer in Rats

**DOI:** 10.1371/journal.pone.0082910

**Published:** 2013-12-23

**Authors:** Jun-Chao Guo, Jian Li, Ying-Chi Yang, Li Zhou, Tai-Ping Zhang, Yu-Pei Zhao

**Affiliations:** Department of General Surgery, Peking Union Medical College Hospital, Chinese Academy of Medical Sciences/Peking Union Medical College, Beijing, China; University of North Carolina School of Medicine, United States of America

## Abstract

The extremely dismal prognosis of pancreatic cancer (PC) is attributed, at least in part, to lack of early diagnosis. Therefore, identifying differentially expressed genes in multiple steps of tumorigenesis of PC is of great interest. In the present study, a 7,12-dimethylbenzanthraene (DMBA)-induced PC model was established in male Sprague-Dawley rats. The gene expression profile was screened using an oligonucleotide microarray, followed by real-time quantitative polymerase chain reaction (qRT-PCR) and immunohistochemical staining validation. A total of 661 differentially expressed genes were identified in stages of pancreatic carcinogenesis. According to GO classification, these genes were involved in multiple molecular pathways. Using two-way hierarchical clustering analysis, normal pancreas, acute and chronic pancreatitis, PanIN, early and advanced pancreatic cancer were completely discriminated. Furthermore, 11 upregulated and 142 downregulated genes (probes) were found by Mann-Kendall trend Monotone test, indicating homologous genes of rat and human. The qRT-PCR and immunohistochemistry analysis of CXCR7 and UBe2c, two of the identified genes, confirmed the microarray results. In human PC cell lines, knockdown of CXCR7 resulted in decreased migration and invasion. Collectively, our data identified several promising markers and therapeutic targets of PC based on a comprehensive screening and systemic validation.

## Introduction

Pancreatic cancer (PC) is a human solid malignant tumor with very poor prognosis [1]. Several clinical and pathological factors for PC have been identified, including T stage, lymph node/distant metastasis, carbohydrate antigen (CA) 19-9 level and perineural/intraneural invasion; however, the factors affecting the prognosis of PC remain to be clarified [2], [3], [4], [5]. In addition, molecular events involved in the pathogenesis and progression of PC, such as Kras mutation, have been discovered as hot spots [1]. However, more research is required identify the cellular factors that affect prognosis.

One of the major drawbacks of the previous studies in human samples and cell lines is the difficulty to collect specimens in all stages of tumorigenesis. Therefore, animal models present unique advantages in this aspect. Chemical inducers of PC include N-nitrosobis(2-oxopropyl)amine, azaserine, and 7,12-dimethylbenzanthracene(DMBA) [6], [7], [8], [9], [10]. These models can induce the entire range of carcinogenesis, through advanced stage and metastasis. DMBA has been widely used in the establishment of rat PC models [11], [12], [13], [14], [15], [16], [17]. In our previous studies, we found that acinar cells can transdifferentiate to ductal cells in the tumorigenesis process in the DMBA-induced PC rat model [18]. Nowadays, the notion and importance of acinar to ductal metaplasia (ADM) have been gradually accepted [19]. Previous studies have examined various features of these PC models, including histological/histochemical features [11], [12], [14], high-fat/high-protein diet as a promoter of carcinogenesis [13], glucose metabolism [15], and alterations in various proteins [16]. One recent investigation performed proteomic analysis in the PC rat model [17]. Thus far, the screening of differentially expressed genes in this model has not been reported.

In the present study, our aim was to screen differentially expressed genes in PC using the DMBA-induced PC rat model.

## Materials and Methods

### Animals

Adult Sprague-Dawley (SD) rats were provided by the Experimental Animal Center, Peking Union Medical College Hospital, Beijing, China. Rats were housed under standard conditions, including a pathogen-free environment and free access to food and drinking water. Twenty-four-hour urine samples were collected with metabolic cages in which only water but not food was provided. The Institutional Animal Care and Use Committee at Peking Union Medical College Hospital specifically approved this study and the use of rats. All efforts were made to minimize suffering.

### Establishment of DMBA-induced PC Model in Rats

The DMBA-induced PC model was established in male SD rats according to our previous method [14]. A total of 75 rats were divided into experimental, control and sham groups. DMBA or NaCl crystals (5 mg) were used for rats in the experimental and control groups, respectively, whereas no agent was applied in the sham group. In experimental and control groups, rats were sacrificed at 7 days, 2 weeks, 1 month and 3 months after implantation. Five rats in the sham group were sacrificed at 1 month. Grouping of all the rats is shown in Table 1.

**Table 1:** Numbers of rats in different groups.

Groups	Time	Number
Experimental	7 days	5
	2 weeks	15
	1 month	15
	3 months	15
Control	7 days	5
	2 weeks	5
	1 month	5
	3 months	5
Sham	1 month	5

### RNA Extraction and Microarray Analysis

Total RNA was extracted from –80°C frozen pancreatic tissue samples collected at all time points using the RNeasy mini kit (Qiagen, Germany) according to the manufacturer’s instructions. Total RNA sample concentration and purity were examined and estimated by optical density measurements at 260/280 nm using a NanoDrop Spectrophotometer and agarose gel electrophoresis. The detailed measurement data and quality evaluation is shown in Table 2. The SuperScript II reverse transcription kit (Invitrogen, USA), Genechip kit (Affymetrix, USA) and One-Cycle Target Labeling and Control Reagents (Affymetrix) were also used in the analyses. Hybridization was performed using the Genechip Rat Expression Set 230 (Affymetrix), according to the manufacturer’s instructions. Gene expression analyses were performed using the Affymetrix Rat Genome 230 A array (Affymetrix, Santa Clara, CA, USA). Sequences used in the design of the array were selected from GenBank, dbEST and RefSeq. Gene chip 230 A contains a total of 15166 probe sets representing approximately 4700 known rat genes and 10460 unannotated expressed sequence tags. The accession number in GEO is GPL1355.

**Table 2:** Total RNA measurement data and quality evaluation.

Sample SerialNunber	RNA Concentration (µg/µl)	A_260_/A_280_	RNA Quantity (µg)	Electrophoresis	Final Evaluation
Norm-pan1	2.448	2.03	489.5	Unqualified	Unqualified
D4001	0.905	2.03	181.1	Qualified	Qualified
D4002	1.776	2.03	355.1	Unqualified	Unqualified
D4003	1.375	2.07	275.0	Qualified	Qualified
D4004	1.910	2.06	381.9	Unqualified	Unqualified
DB7001	0.917	2.04	183.3	Qualified	Qualified
DB7002	2.882	2.02	576.3	Unqualified	Unqualified
DB7003	3.511	1.96	702.1	Qualified	Qualified
DB7004	1.872	2.05	374.4	Qualified	Qualified
D7001	1.849	2.05	369.9	Qualified	Qualified
D7002	2.344	2.04	468.8	Unqualified	Unqualified
D7003	1.932	2.05	386.4	Unqualified	Unqualified
D7004	0.542	1.98	108.5	Qualified	Qualified
D7005	2.168	2.03	433.5	Qualified	Qualified
WB2001	2.229	2.03	445.7	Unqualified	Unqualified
WB2002	3.356	1.99	671.3	Unqualified	Unqualified
WB2004	1.532	2.05	306.3	Qualified	Qualified
WB2005	0.437	2.01	87.4	Qualified	Qualified
W2001	1.175	2.03	235.1	Qualified	Qualified
W2004	2.604	2.00	520.8	Qualified	Qualified
W2005	1.539	2.03	307.9	Qualified	Qualified
W2006	2.451	2.02	490.2	Qualified	Qualified
W2007	1.829	2.01	365.7	Qualified	Qualified
W2009	2.660	2.00	531.9	Qualified	Qualified
W2010	1.425	2.03	285.0	Qualified	Qualified
W2011	0.442	2.00	44.2	Qualified	Qualified
W2012	1.949	2.04	389.8	Qualified	Qualified
W2013	1.413	2.02	282.6	Qualified	Qualified
W2014	2.270	2.02	453.9	Qualified	Qualified
MB1001	3.368	1.95	673.7	Qualified	Qualified
MB1002	1.449	2.00	289.7	Qualified	Qualified
MB1003	2.696	1.99	539.2	Qualified	Qualified
MB1004	2.934	1.98	586.7	Unqualified	Unqualified
M1005	3.510	1.94	701.9	Qualified	Qualified
M1010-2	0.970	1.99	194.0	Qualified	Qualified
M1012	1.387	2.02	277.5	Qualified	Qualified
M1013	1.727	2.04	345.4	Qualified	Qualified
M1014	1.983	2.01	396.6	Qualified	Qualified
M1015	2.701	1.99	540.2	Qualified	Qualified
M3001	3.039	1.98	607.9	Qualified	Qualified
M3002	1.624	2.02	324.9	Qualified	Qualified
M3004	0.891	1.98	89.1	Qualified	Qualified
M3005	1.437	2.02	287.4	Qualified	Qualified
M3006	0.432	2.02	21.6	Qualified	Qualified
M3008	0.392	2.02	19.6	Qualified	Qualified
M3009	1.985	2.02	397.0	Qualified	Qualified
M3014	1.466	1.99	293.2	Qualified	Qualified

### Quantitative Reverse-transcription Polymerase Chain Reaction (qRT-PCR)

Total RNA was extracted using TRIzol (Invitrogen). Reverse transcription was performed using the SuperScript II reverse transcription kit (Invitrogen, USA). The TaqTM R-PCR SYBR Green I kit (Takara, Tokyo, Japan) was used for PCR. Amplification steps were as follows: 95°C for 30 s (pre-denaturation), and 40 cycles of 95°C for 5 s and 61°C for 31 s. Beta-actin was designated as the internal control. All the experiments were repeated three times. Primers are listed in Table 3.

**Table 3:** Primers used in this study.

Gene	Primers	length (bp)
β-actin	5′- TCTGTGTGGATTGGTGGCTCT -3′ (forward)	300
	5′- AGAAGCATTTGCGGTGCAC -3′ (reverse)	
CXCR7	5′- GAGCATCTTCTTCCTCGCATG -3′ (forward)	300
	5′- CAACACGGCGTACCATCTTCT -3′ (reverse)	
ATP6v1g2	5′- GAATCTCTGCGTCCCATGACA -3′ (forward)	250
	5′- CCCCATCACCACGACATACAT -3′ (reverse)	
UBe2c	5′- TTCAAAGCAGGTCTCCAACCA -3′ (forward)	400
	5′- CAGCTCAGAAACCACGGAGAA -3′ (reverse)	

### Immunohistochemistry and Staining Evaluation

Antibodies against CXCR7 and UBe2c were purchased form R&D and Abnova, respectively, and the PowerVisionTM two-step staining kit (PV-9000) was from Beijing Zhongshan Biotech Co., China. Briefly, sections were mounted, deparaffinized by xylene and rehydrated by ethanol. Antigen retrieval was performed by microwaving samples for 3 min. After blocking endogenous peroxidase, slides were then incubated overnight at 4°C with the primary antibody at a dilution of 1∶200. Following washing in phosphate buffered saline (PBS), slides were incubated with horseradish peroxidase (HRP)-labeled secondary antibody for 30 min at room temperature. Diaminobenzidine was used as a chromogen. Finally, slides were counterstained with hematoxylin. Brown coloration in the cytoplasm or nucleus was defined as a positive signal. The ratio percentage of staining positive stained cells more than 50% was classified as a the strong positive signal.

### Cell Culture and Knockdown of CXCR7

Two human pancreatic cancer cell lines (PANC-1 and SU86.86) were kind gifts from Professor Helmut Freiss, Heidelberg University, Germany [20], [21]. Cells were cultured in RPMI 1640 medium and DMEM medium. with 10% fetal bovine serum (Invitrogen) at 37°C in a humidified atmosphere of 5% CO_2_. Cells seeded in 6-well plates were transfected with CXCR7 or control (scramble) small interfering RNA both at a concentration of 40 nM using Lipofectamine RNAiMax (Invitrogen), according to the manufacturer’s instructions. The CXCR7 RNAi was purchased from Invitrogen and the sequence is as follows: AGAUGUAGCAGUGCGUGUCAUAGCC.

### Western Blotting

Cells were washed with PBS and collected by scraping into individual tubes. Proteins were extracted according to protein extraction protocols, and protein concentrations were determined using the Pierce BCA protein assay kit (Thermo Scientific, Meridian Rd, Rockford). Protein extracts (80 µg/lane) were electrophoresed on 10% polyacrylamide gels (SDS-PAGE) followed by transfer to PVDF membranes and blocking with 5% non-fat dry milk for 2 h. Membranes were incubated with primary antibody against CXCR7 (Abgent, San Diego, CA) overnight at 4°C. Secondary antibody (anti-rabbit IgG) was incubated for 1 h at 37°C. Blots were washed three times with PBS, exposed to chemiluminescence reagents (Merck Millipore, Darmstadt, Germany), and exposed to photographic film. All experiments were performed in triplicate.

### Cell Migration and Invasion Assays

Transwell inserts (8.0 µm pore size) (CORNING, Chelmsford St) were used for migration and invasion assays. The lower chamber was filled with 500 µL of RPMI 1640 medium or DMEM medium with 10% FBS. PANC-1 and SU86.86 cells were resuspended in the migration medium (serum-free RPMI1640 or DMEM) and 4×10^5^ PANC-1 or SU86.86 cells in 200 µL of medium were added to the upper chamber after being washed twice with PBS. After 24 h of incubation at 37°C, the cells on the upper surface of the membrane were gently and carefully scraped out using cotton tips. The migrant cells that were adherent to the lower surface of the membrane were fixed in 10% formalin at room temperature for 30 min, and then stained with H&E (hematoxylin and eosin stain). The results of the cell migration assay were evaluated by counting the number of cells on the lower surface of the membrane under an inverted microscope in five different fields at a magnification of ×200.

For cell invasion assays, the undersurface of the membrane was coated with ECM gel (Sigma-Aldrich, Saint Louis, MO, USA) in PBS for 5 h at 37°C. PANC-1 or SU86.86 cells (8×10^5^ cells) in serum-free RPMI 1640 medium or DMEM medium were seeded onto ECM gel-coated upper chambers, according to the manufacturer’s instructions. Cells that had crossed the ECM and passed through the pores of the filter were counted in five fields at ×200. The results are representative of three different experiments.

### Statistical Analyses

For microarray results, background correction and normalization were performed. One-way analysis of variance (ANOVA) and Student-Newman-Keuls (SNK) tests were used to detect statistically differentially expressed genes between different groups. In bioinformatic analysis, differentially expressed genes were subjected to GO analysis hierarchical clustering, principal component analysis, SOM and Mann-Kendall test. The Student-t and Chi-square tests were applied to show differences of measurement and categorical data. The statistical software package SPSS13.0 (SPSS Inc., Chicago, Ill) was used. A statistically significant *P* value was defined as *P*<0.05.

## Results

### Successful Establishment of DMBA-induced PC Model in Rats

**Figure 1:**
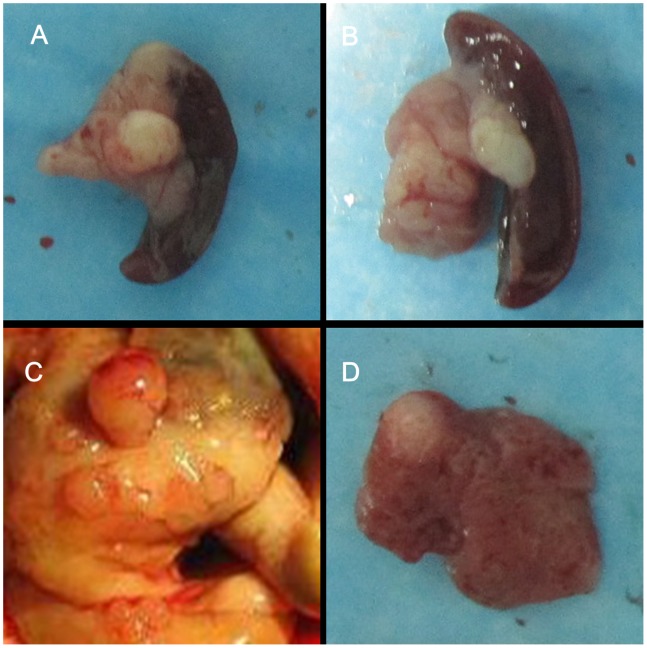
Pancreatic tumor formation and metastasis in the DMBA-induced PC rat model. (A) Pancreatic tumor of 1-month group. (B) Pancreatic tumor of 3-month group. (C) Small intestine metastasis nodules of pancreatic tumor. (D) Liver metastasis nodules of pancreatic tumor.

Acute pancreatitis was observed in rats (7 days after implantation) in both the experimental and control groups. Pancreatic intraepithelial neoplasia (PanIN) was present in 6 out of 15 rats (40%) in the experimental group 2 weeks after implantation, whereas no PC was found in rats in the control group. One month and 3 months after implantation, 12 out of 15 (80%) and 14 out of 14 (100%) rats in the experimental group, respectively, developed PC (Fig. 1A, 1B). Four rats in the 3-month group developed small intestine metastasis or liver metastasis (Fig. 1C, 1D). However, PC was absent in rats in the control groups. The PC formation rate in the experimental group was higher than that in the control group (*P*<0.05). One rat died before the endpoint (3 months), causing a mortality rate of 6.7% (1/15).

**Figure 2:**
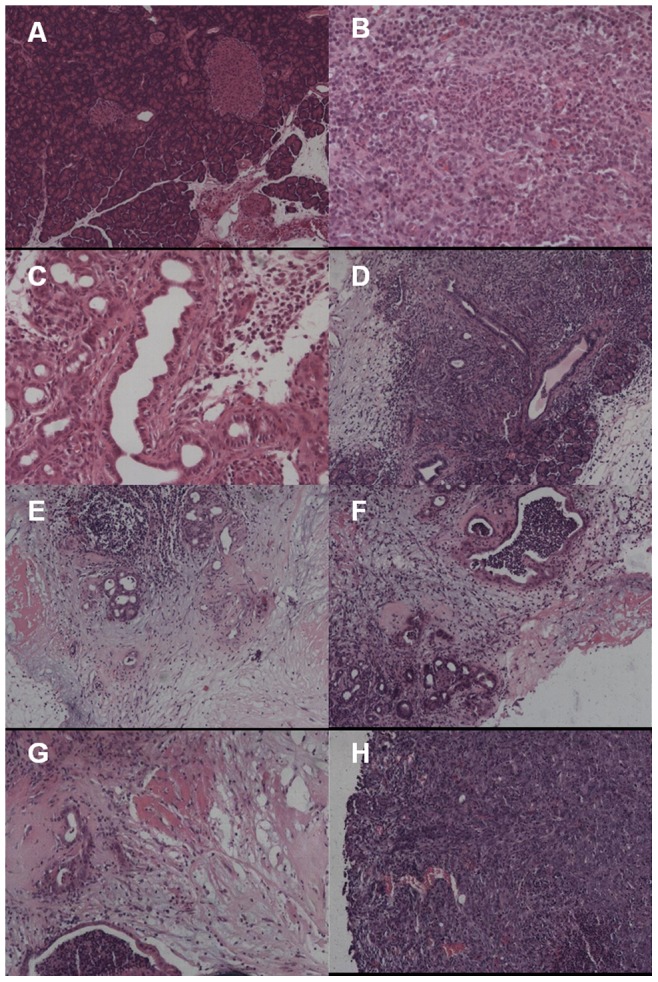
Histological changes in different stages of the DMBA-induced PC rat model. (A) Normal pancreas (original magnification ×60). (B) Acute pancreatitis (original magnification ×150). (C) Chronic pancreatitis (original magnification ×150). (D) PanIN 1a (original magnification ×60). (E) PanIN 2 (original magnification ×150). (F) PanIN 3 (original magnification ×60). (G) Well-differentiated PC (original magnification ×60). (H) Poorly-differentiated PC (original magnification ×60).

Histological changes were evaluated using HE staining and light microscopy. Normal pancreas (Fig. 2A), acute pancreatitis (Fig. 2B), chronic pancreatitis (Fig. 2C), PanIN 2a (Fig. 2D), PanIN 2 (Fig. 2E), PanIN 3 (Fig. 2F), well-differentiated PC (Fig. 2G) and poorly-differentiated PC (Fig. 2H) were observed.

### Differentially Expressed Genes in Rat DMBA-induced PC Model

**Figure 3:**
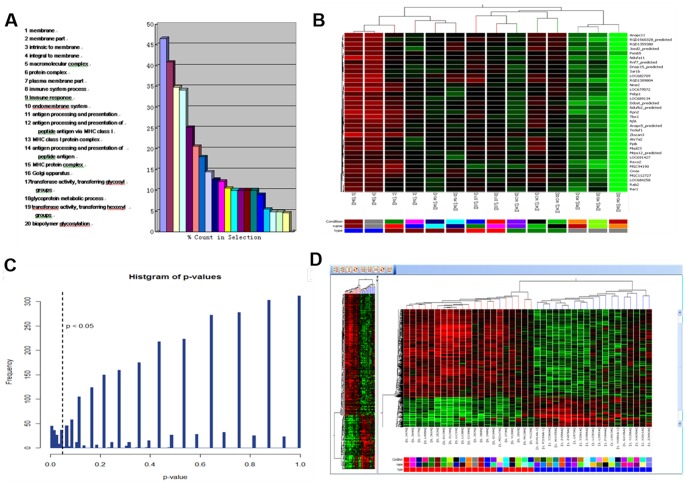
Microarray results. (A) GO classification showing genes involved in different molecular function categories. (B) Two-way hierarchical clustering analysis. (C) *P* values in Mann-Kendall trend Monotone test. (D) Homologous genes in rat and human at different stages of the rat model.

A total of 661 differentially expressed genes were detected in normal pancreas from the control group and the experimental groups (Table 4). According to GO classification, the genes were involved in various functional categories and multiple biological processes, including gas transport, carbohydrate metabolic process, antigen processing and presentation (Fig. 3A). By two-way hierarchical clustering analysis, normal pancreas, acute and chronic pancreatitis, PanIN and early and advanced pancreatic cancer samples were completely discriminated (Fig. 3B). There were 11 upregulated genes (probe) and 142 downregulated genes (probe) by Mann-Kendall trend Monotone test (*P*<0.05) (Fig. 3C). The top 20 upregulated and downregulated genes in the DMBA 3 month group whose expression levels were changed by two-fold compared with the control group (*P*<0.01) are shown in Table 5. In addition, homologous genes in rat and human at different stages of the rat model were successfully screened (Table 6, Fig. 3D).

**Table 4:** Differentially expressed genes in different groups of rats (SNK test).

	Normal	DMBA-7d	DMBA-2w	DMBA-1m	DMBA-3m
Normal	–	1765*	2815*	4826*	2435*
DMBA-7d	1765*	–	413*	1002*	566*
DMBA-2w	2815*	413*	–	530*	393*
DMBA-1m	4826*	1002*	530*	–	947*
DMBA-3m	2435*	566*	393*	947*	–

* *P*<0.05.

**Table 5:** List of upregulated and downregulated genes in DMBA-3 month group whose expression levels were changed by more than 2-fold compared with the control group (*p<0.01*).

Top 20 Genes	
**Upregulated:**	CXCR7, Clcn3, Kras, cyclinB1, nm23, ATP6v1g2, SMAD4, Acf, Adam6, Cyb5b, FGFR3, Mnd1, Kifc1, Car8, Ptpla, EGFR, Park2, c-Myc,Cadps2, Etv5
**Downregulated:**	Emb, Gabbr1, Zdhhc8, CDKN2a, CXCL10, TP53,Gbp2, Tnfsf9, Dnase1l1, Fgr, Vcan, Lyz,Plod2, Stat5a, Smarca2, Cspg4, Rgs14, Ldb3, Bcl2a1, CCR5

**Table 6:** Homologous genes in rat and human at different stages of rats (SNK test).

		Normal	DMBA-7d	DMBA-2w	DMBA-1m	DMBA-3m
**Up**						
	Normal	–	18*	42*	85*	83*
	DMBA-7d	18*	–	18*	29*	18*
	DMBA-2w	42*	18*	–	27*	34*
	DMBA-1m	85*	29*	27*	–	45*
	DMBA-3m	83*	18*	34*	45*	–
**Down**						
	Normal	–	25*	48*	145*	109*
	DMBA-7d	25*	–	17*	12*	10*
	DMBA-2w	48*	17*	–	58*	78*
	DMBA-1m	145*	12*	58*	–	65*
	DMBA-3m	109*	10*	78*	65*	–

* *P*<0.05.

### Validation of Differentially Expressed Genes

**Figure 4:**
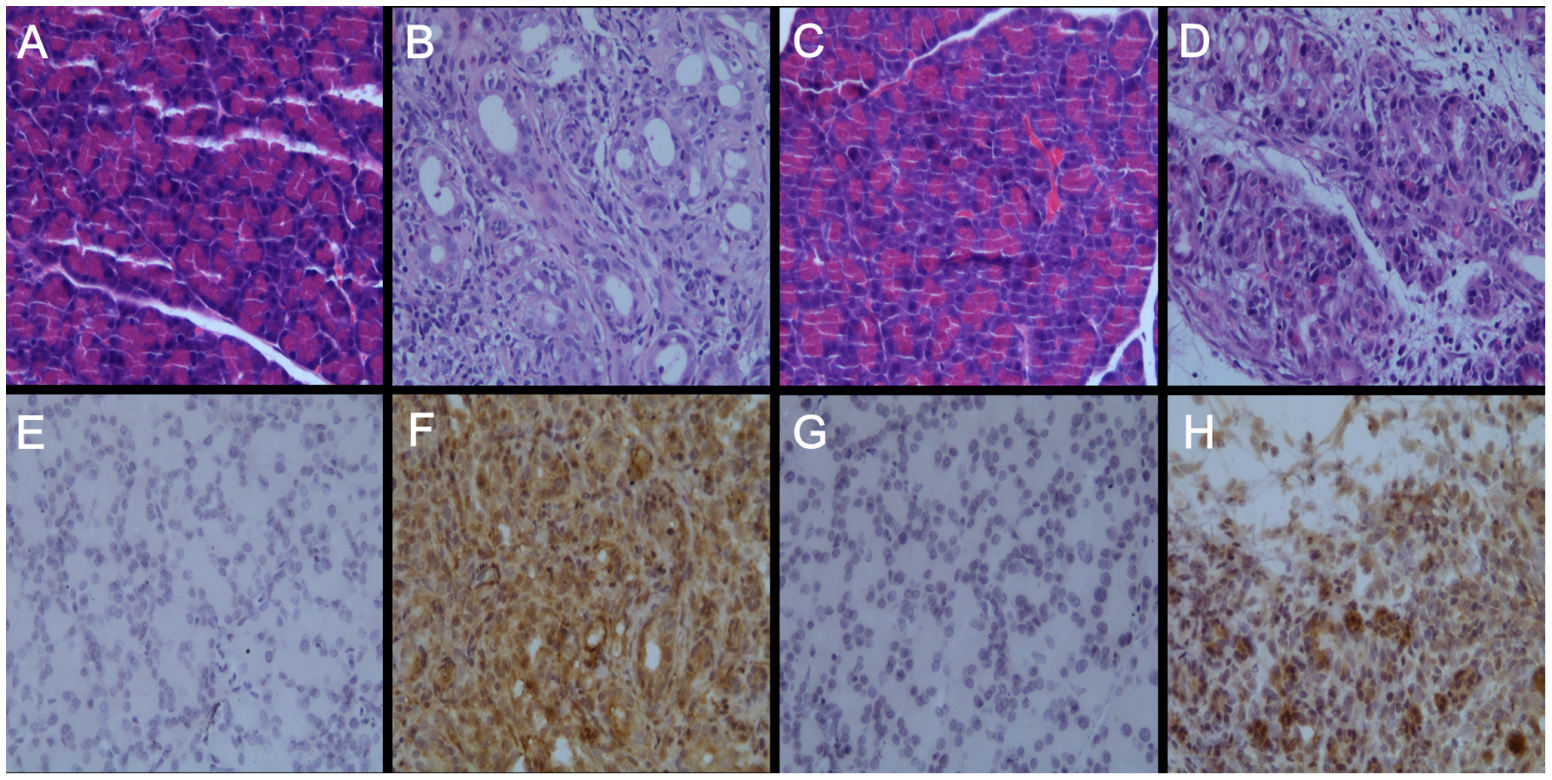
Expression of CXCR7 and UBe2c in DMBA-induced PC rat model (3-month) and normal rat pancreas in control groups. (original magnification ×200). (A),(C) Normal rat pancreas,HE staining. (B),(D) Rat PC,HE staining.(E),(G) Normal rat pancreas,CXCR7 immunohistochemistry staining.(F),(H) Rat PC,Ube2c immunohistochemistry staining.

We selected three of the differentially expressed genes, CXCR7, ATP6v1g2 and UBe2c, for further examination. First, we examined their expression profiles by qRT-PCR. The ΔCt values of CXCR7 gradually decreased, along with prolonged DMBA treatment (*P*<0.05) (Table 5), indicating upregulated expression. There were no significant differences for ATP6v1g2 and UBe2c (*P*>0.05) (Table 7). Using immunohistochemistry, through a pathologist’s diagnosis and confirmation, significantly enhanced CXCR7 and UBe2c expression were detected, along with the progression of PC in rats (*P*<0.05) (Table 8, Figs. 4).

**Table 7:** Validation of some differentially expressed genes by qRT-PCR (ΔCt value).

Genes	Normal	DMBA-7d	DMBA-2w	DMBA-1m	DMBA-3m	*P* value
UBe2c	8.55±0.98	8.02±1.67	8.93±0.55	8.09±0.81	8.26±0.88	0.814
ATP6v1g2	11.55±0.76	10.70±2.60	12.46±1.25	11.49±0.09	11.45±0.46	0.574
CXCR7	7.65±0.65	7.45±0.16	7.10±0.87	6.56±0.22	6.06±0.29	**0.046**

**Table 8:** Validation of some differentially expressed genes by Immunohistochemistry.

		Normal	DMBA-7d	DMBA-2w	DMBA-1m	DMBA-3m	*P* value
**CXCR7**							**0.006**
	Negative	4	4	5	1	0	
	Positive	0	0	1	3	2	
	Strongly positive	0	0	0	2	4	
**UBe2c**							**0.003**
	Negative	4	4	6	0	1	
	Positive	0	0	0	4	4	
	Strongly positive	0	0	0	2	1	

### Decreased Migration and Invasion after Transfection of CXCR7 siRNA in PANC-1 and SU86.86 Cell Lines

**Figure 5:**
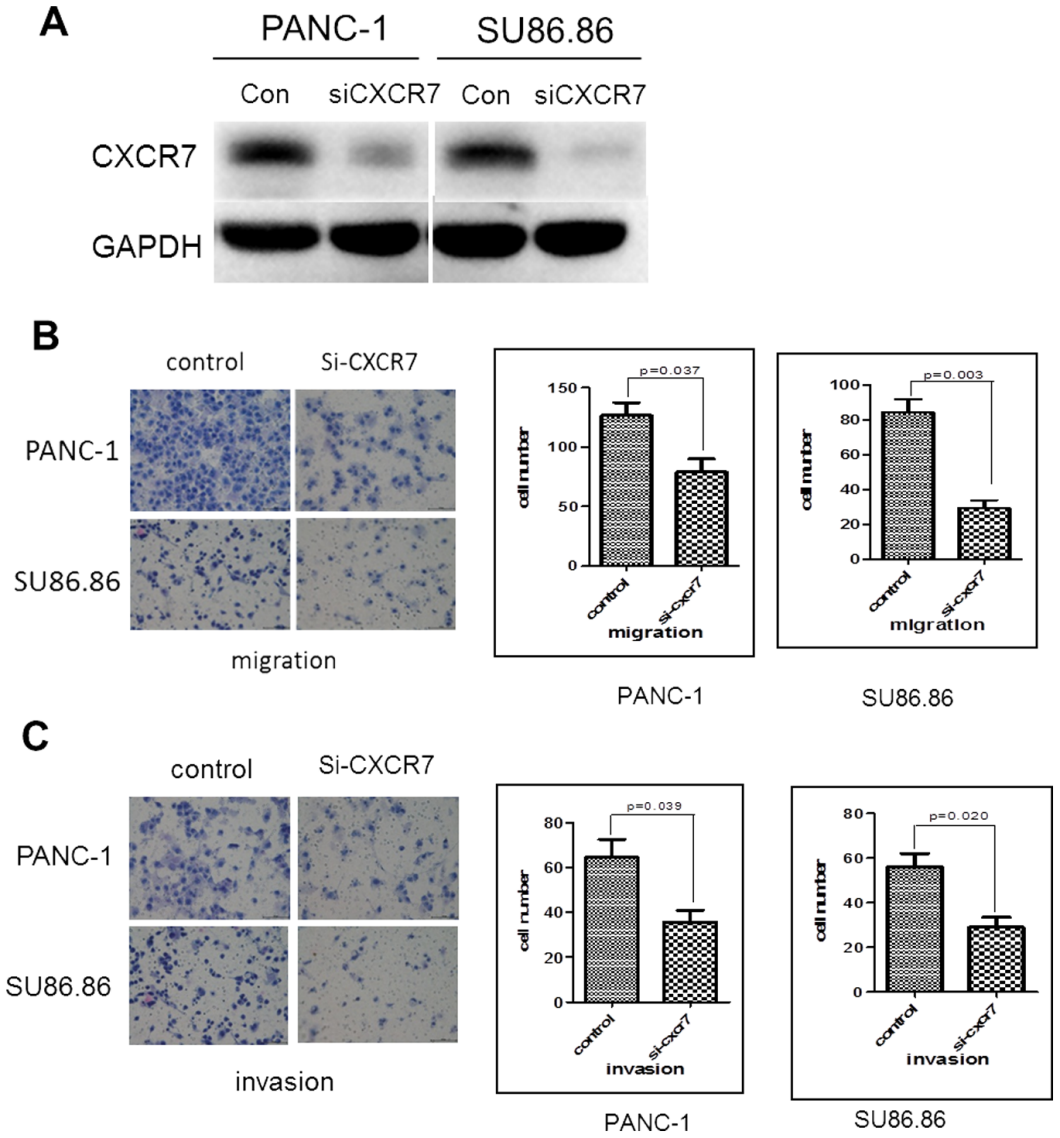
Knockdown of CXCR7 resulted in decreased migration and invasion in human PC cell lines. (A) Knockdown of CXCR7 by siRNA transfection. (B) Migration after transfection of CXCR7 or control siRNAs. (C) Invasion after transfection of CXCR7 or control siRNAs.

Expression of CXCR7 was successfully downregulated in the two human PC cell lines, PANC-1 and Su86.86 (Fig. 5A), after transfection with CXCR7 siRNA, compared to transfection with control siRNA. Significant differences in cell migration and invasion were observed in CXCR7 siRNA transfected cells compared to control cells (P<0.05) (Figs. 5B and 5C).

## Discussion

Various alterations in molecular pathways have been known to play an important role in the initiation and progression of PC [1]. However, one of the complications in studies based on human samples is that features of each disease phase cannot be easily displayed. In this aspect, animal models carry a unique advantage. In the field of PC research, major models have included DMBA-induced and transgenic models [11], [12], [13], [14], [15], [16], [17], [22]. In studies of the DMBA-induced rat model, different DMBA doses and rates of PC development have been reported [11], [12], [13], [14], [15], [16], [17]. The current study, which was based on our previously reported method [14], achieves satisfactory PC induction results. Therefore, these findings indicate successful establishment of the model.

Microarrays have been widely applied in the screening of differentially expressed genes in PC [23], [24], [25], [26]. However, this technique has not been used in the DMBA-induced PC model. The present study examined the gene expression profile in this PC rat model. Affymetrix microarray analysis identified a total of 661 genes that were differentially expressed in normal pancreas from the control group and the experimental groups. These genes included critical facilitators of important functions during pancreatic tumorigenesis [27], such as Kras, CDKN2A, SMAD4, and TP53. These genes were upregulated or downregulated during pancreatic tumor formation duration. GO classification, two-way hierarchical clustering analysis and Mann-Kendall trend Monotone test further identified genes, including nm23, cyclin B1 and FGFR3. A previous study showed that nm23 was associated with metastatic potential of human PC cell lines [28]. In the current study, we showed that nm23 expression was significantly different between normal pancreas and the DMBA 1 month group, suggesting that the molecule might also be involved in the early stage of pathogenesis of PC. Little data is available concerning the roles of cyclin B1 and FGFR3 in PC [23], [29]. Thus here, we provide further evidence that substantiates their involvement in this disease. Further investigations on the detailed mechanisms underlying their involvement in PC are required.

Expression patterns of three differentially expressed genes, CXCR7, ATP6v1g2 and UBe2c, were evaluated by qRT-PCR and immunohistochemical staining. The results showed gradually upregulated CXCR7 expression at the mRNA level, and enhanced positive expression of CXCR7 and UBe2c, along with prolonged DMBA treatment. These findings mostly followed the microarray data. CXCR7 was previously demonstrated to have an impact on growth, apoptosis, invasion/metastasis and prognosis in many cancers [30], [31], [32], [33]. A recent study revealed that CXCR7 promoted cell growth in PC [34]. However, its prognostic significance in PC remains controversial [35], [36]. In the rat model and human cell lines used in our experiments, the association of CXCR7 and progression as well as invasive proclivity of PC was indicated. Therefore, further mechanistic studies for the molecular factors involved in PC worth are warranted.

UBe2c was established to be associated with growth and apoptosis inhibition in cancer cells, thus playing a role in tumorigenesis and showing potential to be a biomarker of both diagnosis and prognosis [37]. However, UBe2c was not previously investigated in PC. In the present study, we found enhanced positive expression of UBe2c along with prolonged DMBA treatment, suggesting its potential role in progression of PC. Notably, UBe2c mRNA expression was not significantly altered, indicating that post-transcription regulation might be involved in changes of its expression. Detailed mechanistic data underlying this activation is required.

In summary, our data identified several potential markers of PC based on a comprehensive screening and further verification.
